# Zinc Absorption from Micronutrient Powders Is Low in Bangladeshi Toddlers at Risk of Environmental Enteric Dysfunction and May Increase Dietary Zinc Requirements

**DOI:** 10.1093/jn/nxy245

**Published:** 2019-01-09

**Authors:** Julie M Long, Prasenjit Mondal, Jamie E Westcott, Leland V Miller, M Munirul Islam, Mondar Ahmed, Mustafa Mahfuz, Tahmeed Ahmed, Nancy F Krebs

**Affiliations:** 1 University of Colorado School of Medicine, Department of Pediatrics, Section of Nutrition, Aurora, CO; 2 International Center for Diarrheal Disease Research, Bangladesh (icddr,b), Nutrition and Clinical Services Division, Dhaka, Bangladesh

**Keywords:** zinc absorption, micronutrient powders, inflammation, enteropathy, Bangladesh

## Abstract

**Background:**

Environmental enteric dysfunction (EED), a chronic inflammatory disorder of the small bowel, is suspected to impair absorption of micronutrients, including zinc.

**Objective:**

The objective of this study was to compare zinc absorption from micronutrient powder (MNP) over a range of zinc doses in young children screened for EED with use of the lactulose:mannitol ratio (L:M).

**Methods:**

Bangladeshi children aged 18–24 mo, grouped according to high and low L:M (≥0.09 and <0.09, respectively), were randomly assigned to MNP with 0, 5, 10, or 15 mg Zn/sachet (10 subjects per dose per L:M group). Over a day, fractional absorption of zinc was measured from an MNP-fortified meal and from unfortified meals with stable isotope tracers; total daily absorbed zinc (TAZ, milligrams per day) was determined as the primary outcome. Secondary outcomes included investigation of relations of TAZ to intake, to physiologic requirement, and to other variables, including biomarkers of systemic and intestinal inflammation, using nonlinear models. TAZ was also compared with published data on child zinc absorption.

**Results:**

In 74 subjects who completed the study, zinc absorption did not differ by L:M grouping. Most biomarkers of intestinal inflammation were elevated in both L:M groups. For combined L:M groups, mean ± SD TAZ for each MNP dose (0, 5, 10, and 15 mg/sachet) was 0.57 ± 0.30, 0.68 ± 0.31, 0.90 ± 0.43, and 1.0 ± 0.39 mg/d, respectively (*P* = 0.002), and exceeded the estimated physiologic requirement only for the 10- and 15-mg MNP doses. Zinc absorption was notably lower at all intake levels compared with published data (*P* < 0.0001) and was inversely related to serum α-1 acid glycoprotein and to fecal *Entamoeba histolytica* (*P* = 0.02).

**Conclusion:**

Results indicate impaired absorption of zinc, which may predispose to zinc deficiency in young children with evidence of enteropathy. These findings suggest that current doses of zinc in MNP may be insufficient to yield zinc-related preventative benefits in similar settings. This study is registered at clinicaltrials.gov as NCT02758444.

## Introduction

Environmental enteric dysfunction (EED) is a disorder of the small bowel which has attracted recent attention as a possible cause of growth faltering, immunity impairment, and micronutrient deficiencies in low-resource settings (1–3). Alterations in small bowel morphology and physiology that occur with EED, including villous atrophy and lymphatic infiltration into the lamina propria and epithelium, may result in malabsorption of various nutrients, including zinc (4). An ongoing challenge, however, is the actual diagnosis of EED with noninvasive biomarkers. No universally accepted case definition or diagnostic criteria currently exists for EED, and the disorder may exist without apparent clinical signs or symptoms.

Based on the lactulose:mannitol ratio (L:M), EED has been reported to be present in ∼44% of children aged <5 y living in an urban slum community (5). The L:M indicates increased gut permeability by lactulose uptake and malabsorption by reduced mannitol absorption, both features of EED. Although often used to identify EED in the field, limitations of the L:M have been highlighted (6), and efforts to identify EED are ongoing (7–9).

Zinc absorption occurs primarily in the proximal small intestine by a saturable transport process, and structural aberrations of the proximal small bowel, as seen with EED, may impair zinc absorption (1, 10, 11). In previous studies of children from low-resource settings, where the inflammatory burden is high, lower than expected zinc absorption was reported, but no studies to date have prospectively linked EED with zinc absorption (12–15). Subclinical zinc deficiency is common in low-resource settings for several reasons, including inadequate dietary intake, low bioavailability, age, and possibly a perturbed gastrointestinal tract (12). In Bangladesh, nearly half of all children aged 6–59 mo have been reported to be zinc deficient (16). They regularly consume foods low in zinc and high in phytate, a pattern assumed to result in low bioavailable zinc (17–19).

The WHO recommends point-of-use fortification of foods with a micronutrient powder (MNP) which includes ∼5 mg elemental zinc in populations where the prevalence of anemia is ≥20% for children aged ≤2 y (20). However, MNPs with this amount of zinc have not been shown to impact zinc-sensitive outcomes, for example growth, diarrheal disease, and infections (21–24). With emerging evidence that EED and zinc deficiency may coexist and may indeed exacerbate one another (11, 12), studies are warranted to directly characterize the extent to which zinc absorption may be affected by EED and whether this impacts zinc requirements for children in impoverished settings.

The primary objectives of this study were to measure zinc absorption from MNP with 4 different doses of zinc added to the local foods of Bangladeshi children grouped by urine L:M, as a putative indicator of EED status; to compare the measured zinc absorption between L:M groups as well as to estimated requirements; and to compare absorption results to larger data sets of zinc absorption in children consuming a wide range of zinc intakes (12). Additional objectives included comparison of biomarkers of intestinal and systemic inflammation, and of status for other micronutrients (iron, vitamin A, and vitamin B-12) according to L:M grouping. We hypothesized that children with low L:M would have a more favorable zinc absorption compared with those with high L:M, and that MNP with a dose higher than 5 mg Zn/d would be necessary to meet the estimated physiological requirement for participants with high L:M.

## Methods

### 

#### Study design

This was a single-blind study to measure zinc absorption from MNP over a wide range of zinc doses (0, 5, 10, or 15 mg/sachet) in Bangladeshi children aged 18–24 mo. Participants, caregivers, field staff, and International Center for Diarrheal Disease Research (icddr,b) laboratory personnel were blinded to assigned MNP dose group. An L:M ≥ 0.09 was used as a screening indicator of EED, a figure that had previously been identified as an appropriate criterion by the icddr,b research team (5, 25, 26). Based on L:M analyses, 40 children were enrolled and assigned to the high L:M (≥0.09) and 40 to the low L:M (<0.09) groups. Participants in each L:M group were then randomly assigned to 1 of 4 MNP doses (*n* = 10/dose, **Supplemental Figure 1**). The MNP-fortified test meal and nonfortified meals were extrinsically labeled with different zinc stable isotope tracers. Measurements included total daily zinc intake (TDZ, milligrams per day); fractional absorption of zinc (FAZ) measured by the dual isotope tracer ratio method (27, 28); absorbed zinc (AZ, milligrams) for each meal; and total daily absorbed zinc (TAZ, milligrams per day). Dietary phytate, serum biomarkers of nutritional status and systemic inflammation, and fecal markers of intestinal inflammation were also measured to further evaluate presence of enteropathy beyond L:M. In addition to summary statistics and comparisons among L:M groups and MNP doses, the relation of zinc absorption to zinc intake and other factors were investigated using nonlinear regression models and by comparison with previously published data.

Written, informed consent was obtained from all participants and the study was approved by the icddr,b Research Review Committee and Ethical Review Committee and the University of Colorado Multiple Institutional Review Board. The trial was registered at clinicaltrials.gov as NCT02758444.

#### Participants

Participants were recruited from a poor peri-urban community of Bauniabadh, Mirpur, in Dhaka, Bangladesh, between November 2015 and October 2016. Data collection and sample analyses were completed in October 2017. Dietary patterns of young children in the community typically include a heavy reliance on rice, lentils, and vegetables, and low intakes of meats, liver, and fish (29).

Local Field Research Assistants (FRAs) and health workers identified children of eligible age (18–24 mo) by door-to-door recruiting for the study using a community birth registry. Parents interested in having their child participate in the study gave verbal consent to continue with the study screening.

Screening, including anthropometry and hemoglobin (Hb) measurements (HemoCue 201, Angelholm, Sweden), was undertaken at the local Mirpur health clinic (**Supplemental Figure 2**). Inclusion criteria were absence of apparent health problems that would impair the ability to consume study diet; willingness to consume MNP and to comply with the demands of the isotope studies; length-for-age *z* score between −1.25 and −3.0 based on WHO Child Growth Standards (30); and Hb ≥8 g/dL. Exclusion criteria included chronic illness; diarrhea treated with zinc supplement within 2 wk of screening; and severe acute malnutrition, defined as weight-for-length *z* score <−3 or mid-upper arm circumference <115 mm or edema.

After written informed consent, the child and guardian returned to the Mirpur health clinic within 7 d for L:M testing. FRAs administrated an oral solution of lactulose (250 mg/mL) and mannitol (50 mg/mL) at a dose of 2 mL/kg (maximum of 20 mL) to participants (5). During the subsequent 2-h urine collection (8), FRAs collected sociodemographic data on the participants.

Urine analyses for L:M were completed in the parasitology laboratory at icddr,b (5). The randomly assigned MNP groups were coded A, B, C, and D, rather than by actual zinc dose. Block lists for random assignment (*n* = 8/block) were created at the University of Colorado School of Medicine (UC) by nonstudy personnel using a random number generator in Excel and were provided to the study coordinators at icddr,b.

#### Power and sample size

Power analysis to determine an adequate sample size was based on testing for differences in saturation response model (SRM) parameter values (31) with the model being fit to absorption data from children with EED or without EED. Because SRMs of zinc-absorption data from these populations did not exist, SRM parameters for hypothesized EED and non-EED absorption response curves were derived from modeling of existing data from absorption studies of children in low-resource countries (14, 15). The characteristic difference between these response curves was that zinc absorption increased more slowly with increasing intake for the EED curve compared with the non-EED curve. Monte Carlo simulation was used to determine the statistical power for detecting hypothetical parameter differences. Using α = 0.05, the results indicated statistical power of 0.85 for a total sample size of 80, that is, 40/L:M group (10 subjects/dose × 4 doses).

#### MNP preparations

Four nutrient premixes were prepared containing 12.5 mg Fe, 300 μg vitamin A, 50 mg vitamin C, 5 μg vitamin D, and 150 μg folate, differing only by the amount of zinc (as gluconate) per sachet (0, 5, 10, or 15 mg) (Hexagon Nutrition). The UC laboratory confirmed zinc content before study initiation. Premixes were individually packaged in temperature-, light-, and water-resistant sachets. Other than labeling of A–D, the packaging and appearance of the MNPs were indistinguishable.

#### Description of test meals: preparation and administration

All study meals were prepared on site in the icddr,b food laboratory using standard recipes developed in previous studies to reflect typical diets of young children in the study area (17). Both the MNP-fortified and unfortified meals consisted of rice, dal, and fried green papaya; bananas were provided as a low-zinc snack if the child expressed hunger between meals. Two days before the isotope studies, under the supervision of the research team, participants were given the study meal with assigned MNP to assess meal and MNP tolerance and acceptance. No adverse events were noted throughout the study.

Children were admitted to the Clinical Trials Unit at icddr,b for the feeding study and isotope administration. On day 0 (Supplemental Figure 2), all meals were labeled with zinc sulfate stable isotope tracers and provided to participants; the MNP was mixed into the foods of the second meal. To quantify intake, pre- and postweights of all meals were measured with a food scale to the nearest whole number (Oxo Good Grips food scale). Duplicate diets of the first and third meals were combined and transferred to a zinc-free plastic container. Similarly, a second duplicate diet was made from the MNP meal, and a third from any additional foods consumed outside of the test meals. Duplicate diets were frozen at −20°C until further processing and analyses.

#### Preparation and administration of isotopes

Individual ^67^Zn, ^68^Zn, and ^70^Zn stable isotope doses (Trace Science International) for each participant were prepared in the pediatric nutrition laboratory at UC as previously described (28, 32); assayed for sterility, fungal growth, and pyrogenicity; and hand carried to icddr,b. Doses were refrigerated in a locked office until administration.

Oral administration of aqueous zinc sulfate isotope doses was initiated halfway through meals (28). For each of the 2 meals without MNP, accurately measured amounts of either 100 (0 mg Zn group) or 200 µg ^67^Zn (5, 10, and 15 mg Zn MNP groups) were carefully given by the study coordinators. Similarly, during the meal containing MNP, 150–1000 µg of ^70^Zn were orally administered, according to MNP zinc dose assignment, with isotope doses providing ≤10% of ingested zinc in the meal. At ∼1500 on day 0, a sterile, accurately measured quantity of ^68^Zn (∼500 µg) was intravenously administered via a peripheral vein (28). All dose losses from saliva and blood were collected for subsequent isotopic enrichment analysis to adjust the administered dose.

#### Sample collections

FRAs and health workers assisted guardians in collecting a clean, baseline spot urine sample before isotope administration. A partial stool sample was also collected for routine and microscopic examination including presence of blood, mucus, or worms, and culture and sensitivity testing for enteropathogens was done at the icddr,b diagnostic laboratory immediately after collection. The remainder of the stool was frozen at −20⁰C until analyses for inflammatory markers. On days 3–6, health workers visited participants’ homes to collect morning and evening spot urine samples (∼20 mL each). Samples were stored at −20⁰C until analyses at icddr,b or shipment to UC. All collection materials were zinc-free, and collection procedures were conducted to avoid zinc contamination.

On day 0 before IV isotope administration, ∼5 mL blood was collected and serum stored at −80⁰C at icddr,b until analyzed.

#### Sample analyses

Zinc stable isotope sample purification and analyses were completed at UC. Total zinc concentrations in the diet were measured by flame atomic absorption spectrophotometry (28). Urine samples were purified using a chelation procedure (28) before zinc isotope ratios were measured using inductively coupled plasma MS and converted to percentage of enrichment (33). Dietary phytate samples were freeze-dried and sent to the USDA Agricultural Research Service for analyses of phytic acid phosphorus by a modified ferric precipitation method (34).

Serum inflammatory and nutritional biomarkers were analyzed at the nutrition biochemistry laboratory at icddr,b. Serum α-1 acid glycoprotein (AGP), high sensitivity C-reactive protein, and soluble transferrin receptor were measured by immuno-turbidimetric assay; ferritin and vitamin B-12 by electrochemiluminescence immunoassay using automated immunoassay analyzer (Roche, Cobas e601); serum retinol by HPLC (Shimadzu Corporation); serum zinc by flame atomic absorption spectrophotometry (Shimadzu AA-6501S); and TNF-α by ELISA (ELISA Quantikine Human TNF-α immunoassay kit, R&D Systems).

Fecal inflammatory markers and pathogens were measured at the icddr,b microbiology laboratory using commercial ELISA kits (calprotectin and myeloperoxidase, ALPCO Immunodiagnostics; neopterin, Genway Biotech; α-1 antitrypsin, Immunochrom GmbH; and antibody detection of *Giardia lamblia, Entamoeba histolytica*, and *Cryptosporidium parvum*, Techlab) (35). Microscopic examination of stool samples for ova of common helminths was done within 2 h of collection (36) and samples were cultured for common pathogens (*Vibrio spp*., *Salmonella spp*., *Shigella spp*., and *Campylobacter spp*.) (36).

#### Data calculations and analyses

FAZ was determined using the dual isotope tracer ratio method (27, 28). Dietary zinc (DZ, milligrams) from MNP meals was measured from meal duplicates, and AZ (milligrams) was calculated by multiplication of ^70^Zn FAZ by DZ. Similarly, AZ (milligrams) from non-MNP meals was calculated by multiplying ^67^Zn FAZ by DZ. TDZ (milligrams per day) and TAZ (milligrams per day) were calculated as the sum of the DZ and AZ from all of the meals. Total dietary phytate (milligrams per day) in duplicate diets was used to calculate phytate:zinc molar ratios.

Summary statistics of FAZ, TDZ, TAZ, total dietary phytate, phytate:zinc molar ratios, biomarkers of nutritional status, and biomarkers of systemic and intestinal inflammation were calculated for L:M groups and compared with the use of *t* tests or nonparametric tests when appropriate. In addition, zinc intake and absorption means were compared by group and by dose (ANOVA), and TAZ was compared with the estimated physiologic requirement for this age group (0.74 mg/d) (37). The physiologic requirement represents the amount of zinc to be absorbed to offset endogenous losses plus additional requirements for growth.

The relations of TAZ to TDZ for the L:M groups were analyzed using published (31, 38) nonlinear models designated here as SRMs. Additional comparisons were made to similar modeling of data from healthy 9- to 10-mo-old breastfed infants in Denver who consumed a range of zinc from different complementary foods (28) and to modeling of a larger data set from multiple zinc absorption studies in children with a wider range of ages and zinc intakes (38) (**Supplemental Table 1**). Brief descriptions of the studies used in the larger data set (38) are provided in Supplemental Table 1. Both of these data sets were derived from studies conducted by our research group using stable isotope methods similar to those applied in the current study. The impacts of phytate, Hb, inflammatory markers, and fecal pathogens on zinc absorption were also examined using SRM (38). In all cases the SRMs were fit to the data using nonlinear regression analysis. Data analyses were performed using GraphPad Prism V.7.00 (GraphPad Software) and R statistical software V.3.2.2 (39). A statistical difference or association was defined as that having *P* value <0.05.

## Results

Seventy-four toddlers completed the study. Four children voluntarily exited the study because perceived study demands were too great, and 2 children were dropped because of incomplete isotope administration (Supplemental Figure 1). No statistical differences were observed between baseline anthropometric and demographic measurements of the L:M groups; only the mean L:M differed (**Table 1**). Mean length-for-age *z* score, weight-for-age *z*score, and weight-for-length *z* score were low and consistent with a high prevalence of stunting and wasting, respectively. Most participants in both groups were mild or moderately anemic, with 67% and 72% of participants in the high and low L:M groups, respectively, having Hb between 8.0 and 10.9 g/dL (40) (Table 1).

**TABLE 1:** Baseline demographic and anthropometric data in Bangladeshi toddlers at risk of environmental enteric dysfunction by L:M group^1^

	High L:M (*n* = 40)	Low L:M (*n* = 40)	*P* value
L:M	0.212 ± 0.15	0.058 ± 0.02	<0.0001
Age, mo	19 ± 2	19 ± 2	0.73
Gender, *n*, M/F	21/19	17/23	
Length, cm	77.2 ± 2.0	76.8 ± 2.2	0.40
Weight, kg	9.1 ± 1.1	8.8 ± 0.9	0.19
LAZ	−2.11 ± 0.44	−2.14 ± 0.44	0.76
WAZ	−1.70 ± 0.85	−1.82 ± 0.75	0.51
WLZ	−0.93 ± 1.06	−1.06 ± 0.87	0.55
Hb, g/dL	10.3 ± 1.4	10.5 ± 1.2	0.49

1 High L:M ≥ 0.09, Low L:M < 0.09. Values are means ± SDs unless otherwise noted; group means are compared by *t* test. Hb, hemoglobin; LAZ, length-for-age *z* score; L:M, lactulose:mannitol ratio; WAZ, weight-for-age *z* score; WLZ, weight-for-length *z* score.

None of the measured biomarkers of systemic inflammation (e.g., AGP, high sensitivity C-reactive protein, and TNF-α), fecal inflammatory markers (e.g., α-1-antitrypsin, calprotectin, myeloperoxidase, and neopterin), or markers of nutritional status exhibited statistical differences between the low and high L:M groups (**Table 2**, **Supplemental Table 2**). With the exception of myeloperoxidase, the median of all fecal inflammatory biomarkers exceeded the normal range for both L:M groups. Myeloperoxidase levels were elevated in over a third of participants in both groups. The inflammatory biomarker data generally exhibited nonnormal distributions with positive skew.

**TABLE 2:** Biomarkers of inflammation in Bangladeshi toddlers at risk of environmental enteric dysfunction by L:M groups^1^

	Normal range^2^	High L:M	Low L:M
Serum biomarkers of systemic inflammation^3^			
α-1 acid glycoprotein, mg/dL	50–120	90 (76,142)	89 (70,122)
High sensitivity C-reactive protein, mg/L	<2.8	0.66 (0.26,3.01)	0.66 (0.23,3.19)
TNF-α, pg/mL	<29.4	27 (26,29)	27 (26,30)
Fecal biomarkers of intestinal inflammation^4^			
Calprotectin, mg/dL	<50	128 (93,261)	153 (42,488)
Myeloperoxidase, µg/mL	<2000	1317 (862,2740)	1750 (763,3515)
Neopterin, ng/mL	<70	582 (368,1287)	1000 (367,1543)
α-1-antitrypsin, mg/L	<0.27	0.52 (0.23,1.03)	0.44 (0.21,0.75)

1 Values are medians (interquartile values); no statistical differences observed between groups by Mann-Whitney nonparametric test for any analyte. L:M, lactulose:mannitol ratio.

2 Sources for normal ranges can be found in **Supplemental Table 3**.

3 *n* = 21–24 for biomarkers in the High L:M group; *n* = 27–29 for biomarkers in the Low L:M group.

4 *n* = 33 for biomarkers in the High L:M group; *n* = 27–37 for biomarkers in the Low L:M group.

No statistical differences in the measures of zinc intake and absorption were observed between the L:M groups. It was also noted that L:M was not correlated with other variables. The zinc intake and absorption data for the L:M groups were therefore combined to examine the mean results for each of the MNP doses (**Table 3**). For the combined L:M groups, mean (±SD) zinc consumption from the MNP-fortified meals at each dose was 0.98 ± 0.30, 4.9 ± 2.0, 9.0 ± 3.3, and 13.0 ± 3.8 mg, whereas zinc intake from the other meals of the day was similar for all MNP groups, averaging 1.9 mg. Fractional absorption from the test meal containing the MNP with no zinc was higher (0.19) than was FAZ from the MNP-containing meals, where FAZ maintained a relatively constant value (0.06–0.07) regardless of MNP zinc dose size. In contrast, FAZ from the other meals declined with increasing MNP dose, even though the zinc consumed from those meals was constant. Except for the placebo MNP group, the zinc intake from non-MNP-fortified meals accounted for ∼13–30% of the total daily zinc intake for each MNP dose group. Means of TAZ demonstrated that the estimated physiologic requirement for this age of 0.74 mg/d (37) was met only by the 10 and 15 mg MNP groups, for whom means of daily absorbed zinc were 0.90 ± 0.43 and 1.0 ± 0.39 mg/d, respectively.

**TABLE 3:** Zinc intake and absorption in Bangladeshi toddlers at risk of environmental enteric dysfunction by MNP doses for combined L:M groups^1^

	MNP dose group			
	0 mg (*n* = 19)	5 mg (*n* = 19)	10 mg (*n* = 19)	15 mg (*n* = 17)
MNP-fortified test meal				
DZ, mg^2^	0.98 ± 0.30	4.9 ± 2.0	9.0 ± 3.3	13.0 ± 3.8
^70^FAZ	0.19 ± 0.09^cde^	0.07 ± 0.02^c^	0.07 ± 0.05^d^	0.06 ± 0.03^e^
AZ, mg	0.19 ± 0.11^cd^	0.34 ± 0.17^e^	0.58 ± 0.38^c^	0.77 ± 0.39^de^
Other meals				
DZ, mg	1.8 ± 0.66	2.0 ± 0.83	1.9 ± 0.69	1.8 ± 0.50
^67^FAZ	0.22 ± 0.10^c^	0.18 ± 0.06	0.17 ± 0.06	0.13 ± 0.03^c^
AZ, mg	0.38 ± 0.20	0.34 ± 0.15	0.32 ± 0.15	0.23 ± 0.07
All meals combined				
TDZ, mg/d^2^	2.7 ± 0.93	6.9 ± 2.7	10.9 ± 3.5	14.9 ± 4.1
TAZ, mg/d	0.57 ± 030^cd^	0.68 ± 0.31^e^	0.90 ± 0.43^c^	1.00 ± 0.39^de^
Phy:Zn MR	8.4 ± 3.5^cde^	2.9 ± 0.97^c^	2.1 ± 0.96^d^	1.4 ± 0.67^e^

1 Values are means ± SDs; multiple comparisons in ANOVA were evaluated using Tukey's range test. AZ, absorbed zinc (milligrams per meal); DZ, dietary zinc (milligrams per meal); FAZ, fractional absorption of zinc, L:M, lactulose:mannitol ratio; MNP, micronutrient powder; Phy:Zn MR, phytate:zinc molar ratio; TAZ: total absorbed zinc (milligrams per day); TDZ, total dietary zinc (milligrams per day).

2 All multiple pairwise comparisons between groups in this row were statistically different (adjusted *P* < 0.05); means in a row with a common superscript letter differ, adjusted *P* < 0.05.

**FIGURE 1:**
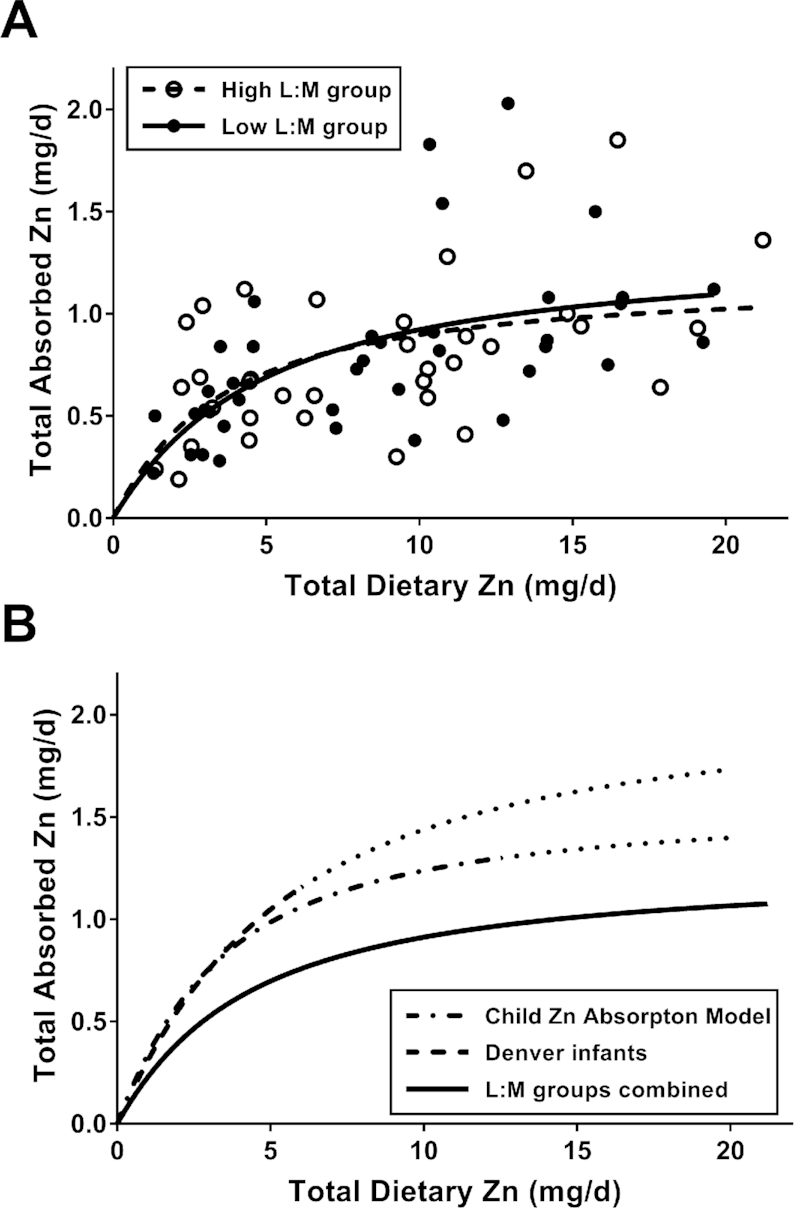
The relation of total absorbed zinc to total dietary zinc intake in Bangladeshi toddlers (A) grouped by high (≥0.09) and low (<0.09) L:M and (B) compared with reference data. (A) The curves show the similar fits of a saturation response model to each L:M group. The parameter data for the curves are presented in Table 4. (B) Comparison of the combined data from (A) to similar modeling of data from a study of breastfed infants in Denver (28) (highest curve) and from the analysis of data compiled from multiple studies of zinc absorption in infants and children (38). The curve representing the latter model shows the prediction for infants of age 19 mo, the mean age of the subjects of this study. For both curves from published analyses, the dashed portion of the curve shows the ranges of the data that were modeled in each case and the dotted portion represents the extrapolation of the curve to cover the range of the current data. L:M, lactulose:mannitol ratio.

The absence of a difference in absorption between the low and high L:M groups was also evident in the SRM relating the absorption data to the intake data (**Figure 1**A). As indicated by the similar regression curves, the resulting parameter estimates were comparable (**Table 4**). The TAZ data for both L:M groups of children exhibited notably lower zinc absorption than that predicted by SRM of healthy 9-mo-old breastfed infants in Denver and for that predicted for 19-mo-olds from SRM of zinc absorption by children in several economically developing countries (Figure 1B) (38). Observed TAZ values were, on average, lower by 0.3 mg/d than the child absorption model predictions (*P* < 0.0001).

**TABLE 4:** SRM parameter estimates for results shown in Figures 1 and 2^1^

Model - predictors of total absorbed zinc	Parameter	Estimated value	95% CI limits	*P* value
Figure 1A				
Total Zn intake (High L:M)	*A_MAX_*	1.17	0.77, 1.57	<0.0001
	*K_T_*	2.79	−0.62, 6.20	0.11
Total Zn intake (Low L:M)	*A_MAX_*	1.33	0.85, 1.81	<0.0001
	*K_T_*	3.99	−0.38, 8.35	0.072
Figure 2				
Total Zn and total phytate intake (Figure 2A)	*A_MAX_*	0.0189	0.0141, 0.0237	<0.0001
	*K_T_*	0.052	0.0085, 0.096	0.020
	*K_P_*	—^2^	—^2^	
Total Zn intake and AGP (Figure 2B)	*A*	8.3	−3.3, 20	0.16
	*E*	−0.42	−0.73, −0.113	0.008
	*K_T_*	3.5	0.040, 7.0	0.048
Total Zn intake and *E. histolytica* (Figure 2C)	*A*	0.078	−0.104, 0.26	0.39
	*E*	−0.87	−1.59, −0.146	0.019
	*K_T_*	3.5	0.75, 6.2	0.013

1 Models described elsewhere (38). 95% CI, 95% confidence interval; *A*, maximal absorption modifier factor; AGP, human α-1 acid glycoprotein; *A_MAX_*, maximal absorption, i.e., absorptive capacity; *E*, maximal absorption modifier exponent; *E. histolytica, Entamoeba histolytica;K_P_*, zinc-phytate binding equilibrium dissociation constant; *K_T_*, zinc-transporter binding equilibrium dissociation constant; SRM, saturation response model.

2 Because of the absence of phytate effect, *K_P_* cannot be estimated.

**FIGURE 2:**
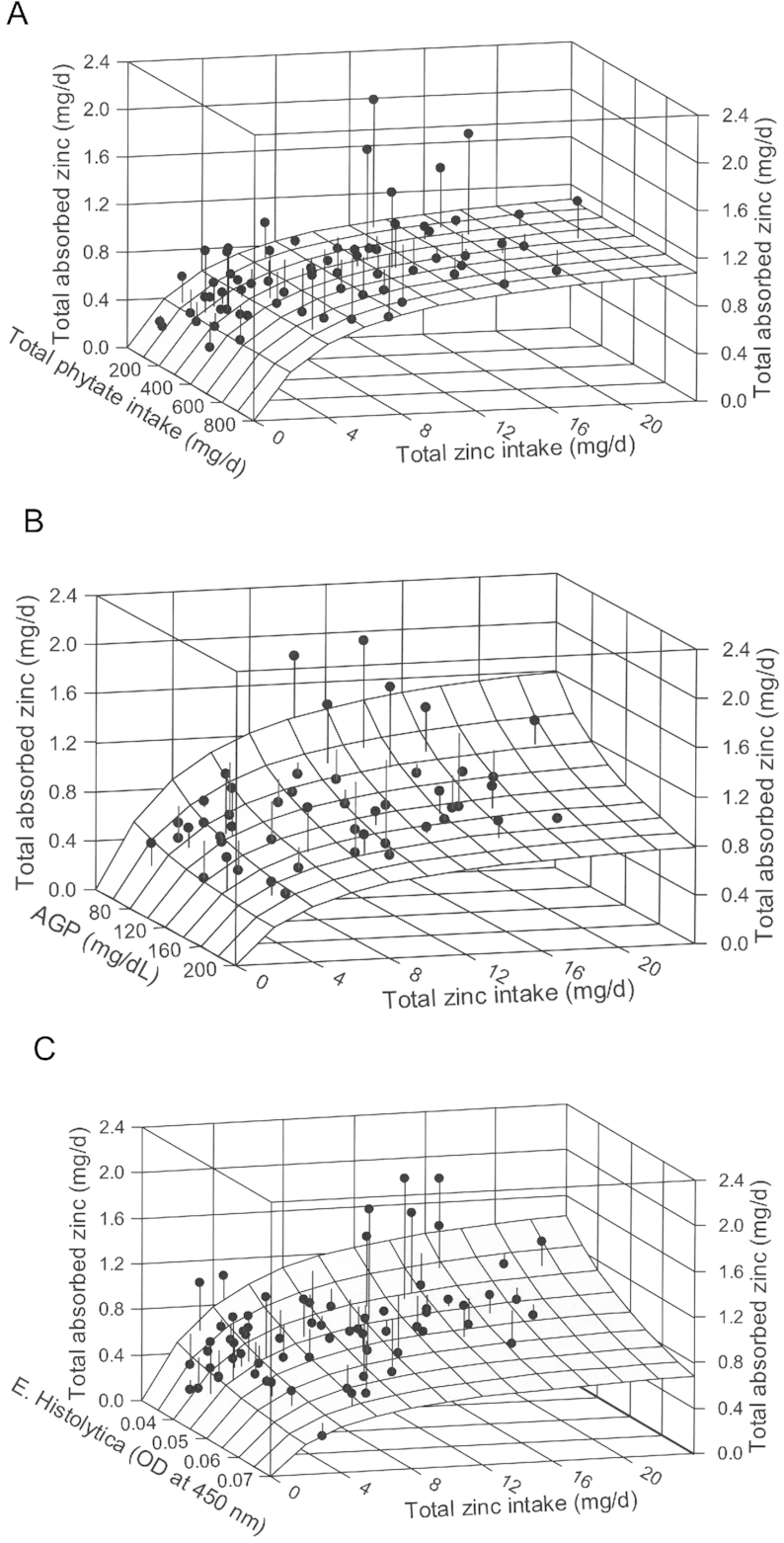
Relations of absorbed zinc to dietary zinc and (A) phytate intake, (B) AGP, and (C) *E. histolytica* in Bangladeshi toddlers. (A) The relation of total absorbed zinc to total dietary zinc and dietary phytate intakes for the combined L:M groups. The surface shows the fit of a saturation response model (38) to the data. The absence of a phytate effect on absorption is apparent from the lack of a slope in the phytate axis. The vertical lines from data symbols to the model surface represent the deviations between data and the model. The parameter data for the models are presented in Table 4. (B) The relation of total absorbed zinc to total dietary zinc intake and serum concentration of AGP, a marker of systemic inflammation, showing decreasing absorption with increasing AGP concentration. (C) The relation of total absorbed zinc to total dietary zinc intake and the presence of *E. histolytica*, an intestinal parasite, showing decreasing absorption with greater infection. AGP, α-1 acid glycoprotein; *E. histolytica, Entamoeba histolitica*; L:M, lactulose:mannitol ratio.

Modeling of TAZ data as a function of both phytate and dietary zinc intake (**Figure 2**A) indicated no effect of phytate on zinc absorption. None of the fecal markers of intestinal inflammation were associated with zinc absorption when controlling for dietary zinc, but the serum concentration of AGP, a marker of systemic inflammation, was negatively associated with zinc absorption (*P* = 0.007; Figure 2B). Of several intestinal pathogens examined, only *E. histolytica* was negatively associated (*P* = 0.02; Figure 2C). Hb was not associated with zinc absorption.

## Discussion

The most noteworthy finding of this study was the low zinc absorption over a wide range of intakes for this group of young children, most of whom had findings consistent with enteropathy. Although the participants were grouped by L:M, a commonly used marker for gut dysfunction, including EED (6), we did not find differences in zinc absorption according to the L:M grouping. However, both groups exhibited strong evidence of intestinal inflammation, considered a hallmark of EED (2, 9, 41), in that a majority of the calprotectin, neopterin, and α-1-antitrypsin data for both L:M groups were above the upper limits of the icddr,b laboratory's proposed normal range. Individual values above the normal range were observed for all inflammatory biomarkers in both groups. Comparison of these children's zinc absorption to that of other populations for similar zinc intakes further supports impaired absorption.

The extent of this impairment predicts that a substantially higher zinc intake than usual dietary recommendations is required to meet estimated physiologic requirements. Only the means of the groups receiving the 2 highest MNP zinc doses, 10 or 15 mg/sachet, had average daily absorbed zinc that met or exceeded the Institute of Medicine's estimated physiologic requirement (37), and even the highest dose did not meet more recent figures proposed (42). In contrast, the healthy breastfed-infant comparison group had total zinc absorption adequate to meet their age-specific physiologic requirement of 0.84 mg/d (37) with average intakes of ∼3 mg/d from fortified cereal or meats (28), that is, with intakes well below the lowest MNP zinc dose of 5 mg in the current study. The findings from the current study may at least partially explain the observations of limited benefits of fortification through MNP with 4–5 mg Zn, in contrast to findings for iron-related outcomes with MNP (23, 24).

Of note, AGP, a marker of systemic inflammation, was negatively associated with zinc absorption, which may reflect more severe enteropathy, including potential bacterial translocation and/or a more pro-inflammatory gut pathogen profile (41). Current understanding of the regulation of zinc absorption does not include a protein analogous to hepcidin, which is stimulated by inflammation and blocks iron transfer into the circulation from the enterocyte (43). Observations from an animal model suggest that the zinc transporter ZIP14, which sequesters zinc in the liver in response to inflammatory stress, may also have a role in intestinal barrier function (44). However, its potential role in zinc absorption under conditions of gut inflammation is unknown. We thus propose that the inverse relations between systemic inflammation and zinc absorption primarily reflect factor(s) in the gut. The negative association between *E. histolytica* on zinc absorption is consistent with this, as its clinical presentation can range from asymptomatic colonization to an invasive mucosal damaging pathogen. Further, it has been reported to be relatively prevalent in young children living in the region of the present study (45).

The absence of a phytate effect over the range of MNP zinc doses is consistent with modeling results of zinc absorption data from stable isotope studies in >200 young children (38). The foods used for the test meals in the current study were typical of the children in this community (17), and the measured phytate content was high in the diet composites. The addition of the fortificant zinc from the MNP resulted in a much lower phytate:zinc molar ratio, for example, <5 for all of the MNP doses above the zero level, and thus diminished the impact of phytate. If these observations are confirmed, they suggest that the impact of the “impoverished gut” as observed in EED may be a more potent driver of zinc deficiency in these high-risk settings than the quality of such plant-based diets (1, 2).

The strengths of this study included the design to prospectively evaluate zinc absorption in children with risk of EED over a wide range of intakes. The limited prestudy exposure to the assigned MNP zinc dose was felt to be insufficient to have any therapeutic effect on gut function. Suboptimal zinc status per se does not result in increased efficiency of absorption (12, 46). Thus, the observed low absorption is likely to reflect habitually impaired absorption capacity. Extrinsic labeling of all meals with stable isotopes and collection of duplicate meals over the entire day permitted the determination of virtually all daily absorbed zinc, and thus enabled comparison with the estimated physiologic requirement and estimation of intakes that would be required to meet it. Such a determination of daily absorbed zinc is more informative than measurement of fractional absorption alone. Additional comparison with typical absorption behavior in populations of young children was possible through the application of an appropriate mathematical model to a large body of absorption data acquired using the same stable isotope tracer techniques. Use of the nonlinear, mechanistic SRM is advantageous in that it more accurately characterizes the saturable response of zinc absorption to dietary intake than linear models and, being multivariate, it permits controlling for intake when evaluating the relation of absorption to other covariates. The consistency of findings of low absorption relative to several reference data supports the conclusion that absorption was compromised in these children who had strong evidence of enteropathy.

The study was not without limitations, the major one being that we were unable to identify a true control group of local participants, either by the primary use of L:M or with multiple other putative markers of EED. Therefore, the hypothesized comparison of absorption in children with and without EED per se was not possible. As discussed above, however, the availability of other references (i.e., estimated physiologic requirement and SRM for this age) provided a suitable means for interpretation of the observed results. The zinc status of the children is also difficult to determine, because the means of serum zinc concentrations for both groups were well within the normal range for the laboratory, despite previous surveys in this community that supported high rates of hypozincemia (16). The limitations of serum zinc as a biomarker are well recognized (42). The propensity for contamination during specimen processing is also high and is suspected to have occurred from unrecognized use of contaminated materials (e.g., gloves) used in the laboratory during our study (supportive data not presented). Because the systemic markers of inflammation, including high sensitivity C-reactive protein, AGP, and TNF, were generally elevated, suppression of serum zinc would have been expected, in contrast to the observed concentrations and in support of contamination of samples.

In summary, the results of this study indicate that over a wide range of intakes and in comparison with several benchmarks, zinc absorption was low in these young children exhibiting evidence of chronic malnutrition. Consistent with other recent reports (6, 8), the L:M did not clearly distinguish participants with or without EED, but multiple biomarkers of systemic and intestinal inflammation were elevated for most participants, indicating that enteropathy was widespread. These findings provide persuasive evidence that impaired zinc absorption contributes to the reported high prevalence of zinc deficiency in this population. Furthermore, they suggest that interventions to prevent zinc deficiency through home fortification in populations at risk of EED will require higher doses of zinc than previously recommended.

## Supplementary Material

nxy245_Supplemental_Files

## Notes

Supplemental Figures 1 and 2 and Supplemental Tables 1–3 are available from the “Supplementary data” link in the online posting of the article and from the same link in the online table of contents at https://academic.oup.com/jn/.

JML and PM contributed equally to this study.

Supported by the Bill and Melinda Gates Foundation OPP 1113134.

Author disclosures: JML, PM, JEW, LVM, MMI, MA, MM, TA, and NFK, no conflicts of interest.

Abbreviations used: AGP, human α-1 acid glycoprotein; AZ, Absorbed zinc; DZ, dietary zinc; EED, environmental enteric dysfunction; FAZ, fractional absorption of zinc; FRA, field research assistants; Hb, hemoglobin; icddr,b, International Center for Diarrheal Disease Research, Bangladesh; L:M, lactulose:mannitol ratio; MNP, multiple micronutrient powder; SRM, saturation response model; TAZ, total absorbed zinc; TDZ, total dietary zinc; UC, University of Colorado School of Medicine.
