# Supplementary material for: Zinc Absorption from Micronutrient Powders Is Low in Bangladeshi Toddlers at Risk of Environmental Enteric Dysfunction and May Increase Dietary Zinc Requirements
Source: J Nutr. 2019 Jan 9;149(1):98–105. doi: 10.1093/jn/nxy245 (PMC6377437; doi:10.1093/jn/nxy245)
Supplement: nxy245_Supplemental_Files [file nxy245_supplemental_files.zip › Supplemental Tables.pdf]

Supplemental Table 1. References and description of subjects, study design and measurements used for comparison to Bangladeshi toddlers

Study	Setting	Subjects	Intervention & Method	Measurements
Krebs et al[1] (1)	Denver, CO	*N=42*, 5- 6 mo old breast-fed (only) infants;	Randomized to first complementary food (CF) meat, multiple-micronutrient fortified infant cereal, or Fe-fortified infant cereal; Zn absorption at 9 mo age	All meals (CF) and human milk of day labeled; dietary phytate Measurements: TDZ, FAZ, TAZ Other: plasma zinc, CRP; anthropometry
Esamai et al (2)	Rural western Kenya	*N=27*, 6 mo old, non-anemic breast-fed infants; CF primarily maize and plant-based	Double-blind, randomized trial of micronutrient powder (MNP), with and without iron or placebo; MNP started at 6 mo of age; zinc absorption studies at 9 mo of age	Weighed duplicate diets including MNP labeled; Measurements: TDZ, FAZ, TAZ Other: serum zinc, AGP, CRP
May et al (3)	Masika, Malawi, isolated village	*N=17*, stunted 3-5 yr olds; habitual diets maize, rice, beans	Studied on habitual diet; repeat isotope studies after 30 days of resistant starch added to habitual diet	Weighed duplicate diets Measurements: TDZ, FAZ, TAZ Other: serum zinc; anthropometry
Chomba et al (4)	Chongwe District, Zambia	*N=60*, 1-5 yr olds; habitual diets primarily maize;	Randomized to biofortified or zinc-fortified or control/local maize; studied on diet x 1 day	Weighed duplicate diets; dietary phytate Measurements: TDZ, FAZ, TAZ Other: plasma zinc, AGP; anthropometry
Sheng et al (5)	Xi-Chou China, Yun-nan province	*N=43*, 19-25 mo olds; habitual diets primarily rice, vegetables	Studied on habitual diets	Weighed duplicate diets; dietary phytate Measurements: TDZ, FAZ, TAZ Other: plasma zinc; anthropometry
Manary et al (6)	Rural Malawi	*N=10*; 2-5 yr olds; maize based diet	Studied on habitual diets	Weighed duplicate diets Measurements: TDZ, FAZ, TAZ Other: plasma zinc, CRP; anthropometry
Manary et al (7)	Blantyre, Malawi Queen Elizabeth Central Hospital	*N=23*; 3-13 yr olds; recovering from tuberculosis or minor injury; well children controls	Children randomized to standard or phytate-reduced corn-plus soy porridge; studied after 3-7 days on assigned porridge	Weighed duplicate diets Measurements: TDZ, FAZ, TAZ Other: plasma zinc, CRP; anthropometry
Kodkany et al (8)	Kineye, Belgaum, Karnataka, India	*N=40*; 22-35-mo olds; vegetarian diet; all subjects iron deficient	Double-blind, randomized assignment to iron and zinc biofortified or control pearl millet; studied on assigned diet x 1 day	Weighed duplicate diets Measurements: TDZ, FAZ, TAZ Other: plasma zinc, CRP; anthropometry

[1]Dataset used in text reference (28); all data sets used in text reference (39)

Supplemental Table 2. Serum biomarkers of nutritional status in Bangladeshi toddlers at risk for environmental enteric dysfunction by lactulose:mannitol ratio (L:M) group[1]

Analyte	Normal range[2]	High L:M	Low L:M
Zn, mg/L	0.65-1.80	0.79 ± 0.40 (22)	0.90 ± 0.46 (29)
Ferritin, ng/mL	20-200	34 ± 22 (24)	31 ± 21 (28)
Soluble transferrin receptor, µg/mL	2.2-6.3	6.2 ± 3.6 (24)	5.3 ± 2.7 (28)
Vitamin B_{12}, pg/mL	264-1215	371 ± 200 (24)	345 ± 180 (28)
Retinol, µg/dL	≥20	32.4 ± 17.5 (21)	32.0 ± 17.6 (28)

[1]Values are presented as mean ± SD (*n*); Group means compared by t-test; no statistical differences ($P < 0.05$) between L:M groups. M, male; F, female.
[2]References for normal ranges can be found in Supplemental Table 3.

Supplemental Table 3. Reference sources for normal ranges reported in this study

Analyte	Normal range	Reference
**Serum biomarkers of nutritional status**		
Zn, mg/L	0.65-1.18	(9)
Ferritin, ng/mL	20-200	(10)
Soluble transferrin receptor, µg/mL	2.2-6.3	(11)
Vitamin B_{12}, pg/mL	264-1215	(12)
Retinol, µg/dL	≥ 20	(13)
**Serum biomarkers of systemic inflammation**		
Alpha-1 acid glycoprotein, mg/dL	50-120	(14)
High sensitivity C-reactive protein, mg/L	0.1-2.8	(15)
Tumor necrosis factor-α, pg/mL	<29.4	(16)
**Fecal biomarkers of intestinal inflammation**		
Calprotectin, mg/dL	<50	(17)
Myeloperoxidase, µg/mL	<2000	(18)
Neopterin, ng/mL	<70	(19)
α-1-antitrypsin, mg/L	<0.27	(20)

References

1. Krebs NF, Westcott JE, Culbertson DL, Sian L, Miller LV, Hambidge KM. Comparison of complementary feeding strategies to meet zinc requirements of older breastfed infants. Am J Clin Nutr. 2012 May 30;96:30-5.
2. Esamai F, Liechty E, Ikemeri J, Westcott J, Kemp J, Culbertson D, Miller LV, Hambidge KM, Krebs NF. Zinc absorption from micronutrient powder is low but is not affected by iron in Kenyan infants. Nutrients. 2014;6:5636-51.
3. May T, Westcott C, Thakwalakwa C, Ordiz MI, Maleta K, Westcott J, Ryan K, Hambidge KM, Miller LV, et al. Resistant starch does not affect zinc homeostasis in rural Malawian children. J Trace Elem Med Biol. 2015 Apr;30:43-8.
4. Chomba E, Westcott CM, Westcott JE, Mpabalwani EM, Krebs NF, Patinkin ZW, Palacios N, Hambidge KM. Zinc absorption from biofortified maize meets the requirements of young rural zambian children. J Nutr. 2015 Mar;145:514-9.
5. Sheng XY, Hambidge KM, Zhu XX, Ni JX, Bailey KB, Gibson RS, Krebs NF. Major variables of zinc homeostasis in Chinese toddlers. Am J Clin Nutr. 2006 Aug;84:389-94.
6. Manary MJ, Hotz C, Krebs NF, Gibson RS, Westcott JE, Broadhead RL, Hambidge KM. Zinc homeostasis in Malawian children consuming a high-phytate, maize-based diet. Am J Clin Nutr. 2002 Jun;75:1057-61.
7. Manary MJ, Hotz C, Krebs NF, Gibson RS, Westcott JE, Arnold T, Broadhead RL, Hambidge KM. Dietary phytate reduction improves zinc absorption in Malawian children recovering from tuberculosis but not in well children. J Nutrition. 2000 Dec;130:2959-64.

8. Kodkany BS, Bellad RM, Mahantshetti NS, Westcott JE, Krebs NF, Kemp JF, Hambidge KM. Biofortification of pearl millet with iron and zinc in a randomized controlled trial increases absorption of these minerals above physiologic requirements in young children. J Nutr. 2013 Sep;143:1489-93.

9. Lockitch G. Trace elements in pediatrics. Journal of the International Federation of Clinical Chemistry. 1996 Jun;8:46-8, 50-1.

10. Heil W, Koberstein R, Zawta B. Reference Ranges For Adults And Children: Pre-Analytical Consideration, 2000. Roche Diagnostics, Mannheim. 1999.

11. Choi JW, Pai SH, Im MW, Kim SK. Change in transferrin receptor concentrations with age. Clin Chem. 1999 Sep;45:1562-3.

12. Hicks JM, Cook J, Godwin ID, Soldin SJ. Vitamin B12 and folate. Pediatric reference ranges. Arch Pathol Lab Med. 1993 Jul;117:704-6.

13. West Jr. KP. Vitamin A: Deficiency and interventions. In: Caballero B, Allen L, Prentice A, editors. Encyclopedia of Human Nutrition. 2nd ed. Amsterdam: Elsevier Ltd.; 2006. p. 348-59.

14. Consensus values of the "Deutsche Gesellschaft für Laboratoriumsmedizin", the "Deutsche Gesellschaft für Klinische Chemie" and the "Verband der Diagnostica - Industriee.V.". DG Klinische Chemie Mitteilungen. 1995;26:119-22.

15. Schlebusch H, Liappis N, Kalina E, Klein C. High Sensitive CRP and Creatinine: Reference Intervals from Infancy to Childhood/Hochsensitives CRP und Kreatinin: Referenzbereich für Neugeborene und Kinder. Laboratoriums Medizin. 2002;26:341-6.

16. R&D Systems Inc. Quantikine® ELISA: Human TNF-α Immunoassay. Minneapolis, MN: R&D Systems, Inc.; 2012.

17. ALPCO. Calprotectin ELISA. Salem, NH: ALPCO; 2017.

18. ALPCO. IDK® MPO ELISA. Bensheim, Germany: Immunodiagnostik AG; 2017.

19. Kosek M, Haque R, Lima A, Babji S, Shrestha S, Qureshi S, Amidou S, Mduma E, Lee G, et al. Fecal markers of intestinal inflammation and permeability associated with the subsequent acquisition of linear growth deficits in infants. Am J Trop Med Hygiene. 2013 Feb;88:390-6.

20. Beckmann G, Rüffer A. Mikroökologie des Darmes - Grundlagen, Diagnostik, Therapie. Neuwertiger Zustand: Schlüter; 2000.
