# Supplementary figures and images for: Zinc Absorption from Micronutrient Powders Is Low in Bangladeshi Toddlers at Risk of Environmental Enteric Dysfunction and May Increase Dietary Zinc Requirements
Source: J Nutr. 2019 Jan 9;149(1):98–105. doi: 10.1093/jn/nxy245 (PMC6377437; doi:10.1093/jn/nxy245)

## Supplementary Data

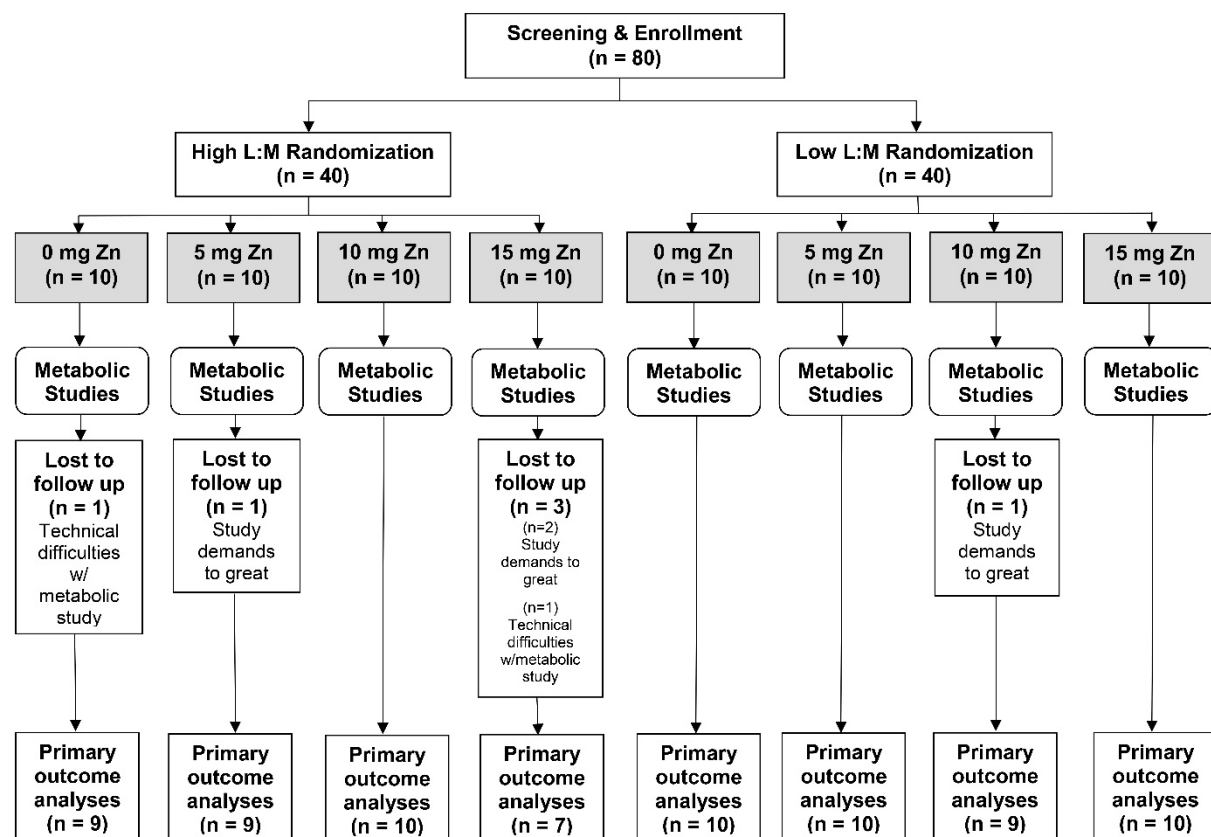

**Supplemental Figure 1** Study consort diagram

Supplement: nxy245_Supplemental_Files [file nxy245_supplemental_files.zip › Supplemental Figure 1.pdf]

## Supplementary data

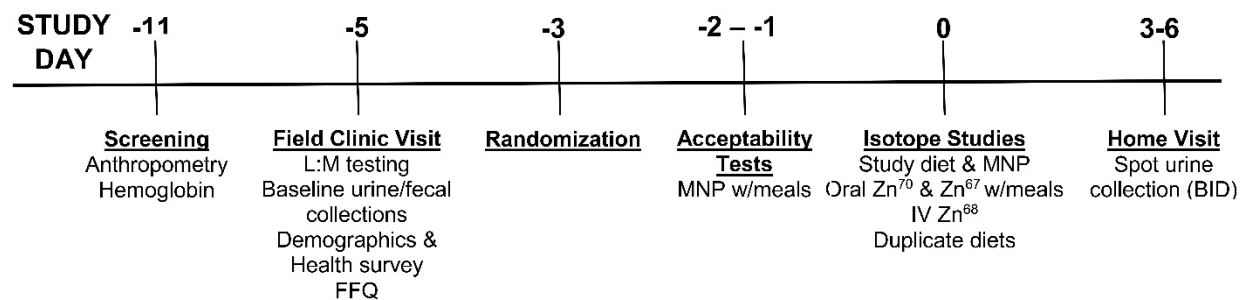

**Supplemental Figure 2** Study design for individual participant

Supplement: nxy245_Supplemental_Files [file nxy245_supplemental_files.zip › Supplemental Figure 2.pdf]
